# Impact of Lifestyle Modification: An Intervention on Newly Diagnosed Diabetics of the Urban Slum of Meerut

**DOI:** 10.7759/cureus.58844

**Published:** 2024-04-23

**Authors:** Komal Anand, Seema Jain

**Affiliations:** 1 Department of Community Medicine, Sarojini Naidu (SN) Medical College, Agra, IND; 2 Department of Community Medicine, Lala Lajpat Rai Memorial (LLRM) Medical College, Meerut, IND

**Keywords:** hba1c, glycosylated hemoglobin, fasting blood glucose levels, lifestyle modification, undiagnosed diabetes

## Abstract

Introduction

Diabetes is a long-term condition that necessitates ongoing medical attention and self-care to prevent immediate complications and minimize the likelihood of long-term issues. Early diagnosis is one of the most important steps for people living with diabetes to take. Public awareness regarding the importance of lifestyle modification in managing type 2 diabetes mellitus is a crucial preventive measure. Despite continuous efforts to raise public awareness, the prevalence of type 2 diabetes continues to increase, with most people overlooking the importance of a healthy lifestyle. Our goal was to assess the impact of lifestyle modification on glycemic control in newly diagnosed diabetic patients.

Materials and methods

A total of 503 adults aged 30 years and above who were nondiabetic or were unaware of their diabetic status were assessed for their fasting blood glucose levels. Individuals identified as diabetic based on their fasting blood glucose levels were subjected to lifestyle modification for a period of three months. Glycemic levels were measured at the beginning and the end of the study period for comparison.

Results

Of the study participants, 7.6% were undiagnosed diabetics with increased blood sugar levels who were unaware of their diabetic status. Mean anthropometric measurements from pre- to postintervention values improved overall. Overall reduction was observed in weight (66.21±12.97 to 63.18±11.48), waist circumference (96.21±13.01 to 91.77±11.82), hip circumference (105.16±11.91 to 103.58±10.88), waist-hip ratio (0.91±0.09 to 0.88±0.08) and body mass index (27.48±6.04 to 26.18±5.30). Significant reductions were observed in the mean glycemic values, including fasting blood sugar (180.19±55.81 to 152.56±45.74) and glycosylated hemoglobin levels (8.61±1.97 to 6.68±1.67).

Conclusion

Lifestyle modification plays a crucial role in managing diabetes, both in preventing its onset and controlling its progression. The present study highlights the importance of early diagnosis and lifestyle interventions in the management of diabetes, thereby stressing the necessity of comprehensive strategies to combat this situation.

## Introduction

Diabetes mellitus is a chronic metabolic condition marked by consistently elevated blood glucose levels and disturbances in the metabolism of carbohydrates, fats, and proteins due to insufficiencies in the production of insulin, its effectiveness, or both. It necessitates ongoing medical attention and self-management education to prevent immediate complications and minimize the risk of long-term issues [1].

The International Diabetes Federation (IDF) Diabetes Atlas reported that the global estimate of individuals with diabetes, including both type 1 and type 2 diabetes, diagnosed and undiagnosed, was 537 million, accounting for 10.5% of the world’s population. This number is predicted to rise to 643 million (11.3%) by 2030 and to 783 million (12.2%) by 2045 [2]. In India, the prevalence of diabetes among individuals aged 20-70 years was reported to be 9.6% in the IDF Report 2021, with a total of 74 million cases of diabetes [2].

The primary treatment goal in diabetes management is glycemic control because it not only improves health outcomes but also reduces the rate of severe complications and comorbidities [3]. Apart from regular oral medications, activities such as healthy eating, increased physical activity, regular monitoring of blood sugars, and self-care practices can lead to better prevention and control [4].

There is compelling evidence indicating that lifestyle changes and medications can significantly reduce the risk of developing type 2 diabetes mellitus. Lifestyle interventions offer a noninvasive method of weight management, aid in glycemic control, and reduce the likelihood of severe complications in type 2 diabetes mellitus patients [3].

Lifestyle modification entails changing longstanding habits, typically related to diet or physical activity, and maintaining these new behaviors for extended periods, which applies to most noncommunicable diseases. Lifestyle modification not only helps in weight loss but also prevents and delays further complications of diabetes [5].

In this study, we aimed to implement such modifications and hypothesized that lifestyle modification would be more effective in enhancing glycemic control in individuals recently diagnosed with type 2 diabetes mellitus. We conducted the current study to assess the impact of lifestyle modification on glycemic control in newly diagnosed diabetic patients.

## Materials and methods

**Figure 1:**
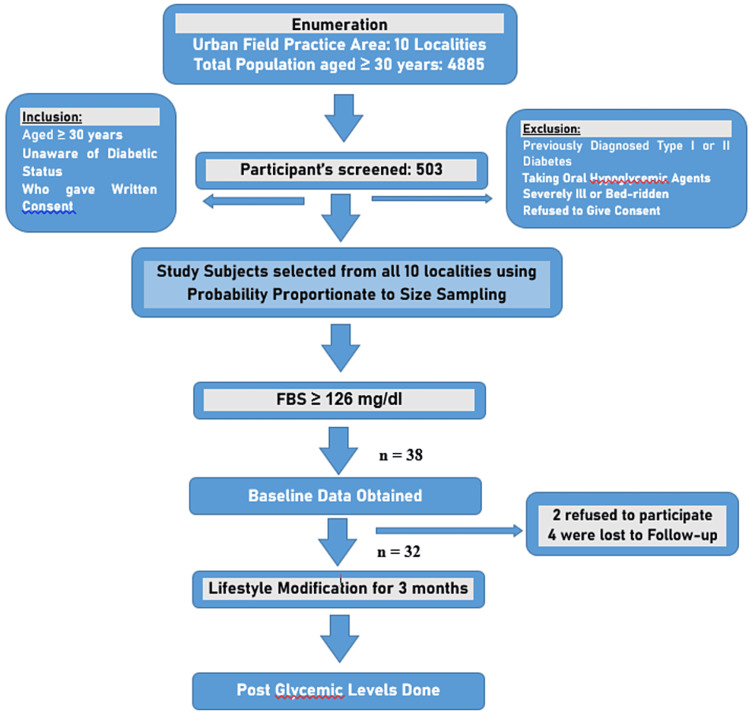
Algorithm showing selection of participants for intervention

The current study was a mixed method type of study that took place in the urban slum area of Meerut. Using the formula, 4pq/l2, where p is the prevalence of undiagnosed diabetes, q is 1-p, and l is the absolute precision, the sample size was calculated. Considering that the prevalence of undiagnosed diabetes is 9% [6] with a 95% confidence interval with an absolute precision of 2.5%, we determined the sample size to be 503. Ethical clearance was obtained from the institutional ethical committee. The study was conducted in the urban field practice area of the Department of Community Medicine in one of the medical colleges in Meerut. This area comprises 10 registered localities. We selected adults aged 30 years and older from each locality using the probability proportionate to size method. From each locality, one house was selected randomly. The random house allocation was done by pencil/pen tip drop method to start the survey. From the center of the selected locality, the pencil/pen was dropped and the house in the corresponding direction towards the tip of the pencil/pen was selected as the first house for the survey. Then house-to-house survey was done picking all the adults aged 30 years and above in the adjacent houses toward the right of the first house till the required sample of adults aged 30 years and above for that locality was covered. We assessed the fasting blood glucose levels of the study subjects. Excluded from the study were those who were previously diagnosed as diabetic and/or were taking oral hypoglycemic agents, were pregnant or lactating, were severely ill, or were bedridden. Individuals who refused to provide written consent were also excluded (Figure 1).

All the study subjects with a fasting blood glucose level > 126 mg/dl and found to have diabetes were informed about the intervention of lifestyle modification. Of the 38 subjects recently diagnosed with diabetes, two were excluded from further participation due to refusing to participate and time constraints. Thus, 36 subjects were enrolled. However, four of these did not participate in follow-up or dropped out from the intervention and therefore were excluded from the analysis.

Study subjects provided written consent to participate in the study and be available for regular follow-ups. All study subjects who gave consent were subjected to lifestyle modification for a period of three months. Glycemic levels were calculated at the start (baseline) and the end and were compared.

We provided lifestyle modification counseling sessions to all recently diagnosed diabetic adults. Counseling sessions were conducted every 15 days for a duration of three months, and anthropometric measurements of the study participants were taken at the beginning and end of the third month.

Lifestyle modification included five key components: 1. Provide a balanced diet chart to each study participant. 2. Increased physical activity in which each study participant was advised to start brisk walking for 30 minutes twice a day. 3. Those taking tobacco and alcohol were advised to stop consuming them. 4. For stress management, regular weekly counseling was done. 5. A routine customized diet chart was given to every participant and were encouraged to stick to that.

We explained all components of the lifestyle intervention during counseling sessions in the local language, supplemented with visual aids such as pictures and videos. We also conducted face-to-face interviews and discussions with individuals or groups. We monitored participants through personal meetings, frequent phone calls, and regular interactions. Each counseling session lasted approximately 10-15 minutes. For feedback from study participants, we recorded daily text messages from each participant. We made voice calls to those who failed to respond to text messages. The calls reminded them to record their daily physical activity status and adherence to diet as advised.

At the end of three months, we again calculated the glycemic levels and anthropometric measurements and compared these results with the baseline values. Data were analyzed using the appropriately trademarked Centers for Disease Control, Epi Info TM 7.2.3.1. We used a paired t-test to compare baseline values and values after three months of intervention.

Operational definitions

Prediabetes

It refers to individuals whose glucose levels do not meet the criteria for diabetes but are too high to be considered normal [7].

Cut-Off Values for Diabetes and Prediabetes

The cut-off values for diabetes and prediabetes are shown in Table 1 [8].

**Table 1:** Diagnostic criteria for diabetes and prediabetes *ADA-American Diabetes Association; **IFG - Impaired Fasting Glucose; ***IGT - Impaired Glucose tolerance; ****FPG - Fasting Plasma Glucose; *****2-h PG-2 hour post load Glucose test (oral glucose tolerance test) plasma glucose; ******HbA1c – Glycosylated Hemoglobin

Parameter	Normoglycemia (mg/dl)		Prediabetes (mg/dL)		Diabetes (mg/dL)
	WHO	ADA*	WHO	ADA*	
****FPG	< 110	< 100	110-125 (IFG**)	100-125 (IFG**)	≥ 126
*****2-h PG	< 140		140-199 (IGT***)		≥ 200
******HbA1c	< 5.7%		5.7-6.4%		≥ 6.5%
Random plasma glucose					≥ 200 (along with symptoms of diabetes)

Criteria for the Diagnosis of Diabetes

The criteria for the diagnosis of diabetes include [7] any one of the following:

 FPG ≥126 mg/dL (7.0 mmol/L). (Fasting is defined as no caloric intake for at least eight hours.)

OR

2-h PG ≥200 mg/dL (11.1 mmol/L) during OGTT (the test should be performed as described by WHO, using a glucose load containing the equivalent of 75 g anhydrous glucose dissolved in water).

OR

HbA1C ≥6.5% (48 mmol/mol).

OR

In a patient with classic symptoms of hyperglycemia or hyperglycemic crisis, random plasma glucose ≥200 mg/dL (11.1 mmol/L).

Physical Activity

WHO recommends a minimum physical activity of 150 minutes per week [9].

Insufficient physical activity: It is defined as the proportion of adults who spent <150 minutes of moderate-intensity physical activity per week OR <75 minutes of vigorous-intensity physical activity per week.

Moderate intensity Physical activity: All those subjects who do moderate-intensity activity that causes a small increase in breathing rate or heart rate such as a brisk walk or carrying light loads for at least 10 minutes continuously.

Vigorous intensity Physical activity: All those subjects who do vigorous intensity activity that causes a large increase in breathing rate or heart rate such as lifting or carrying heavy loads, digging, or construction work for at least 10 minutes continuously.

Body Mass Index (BMI) or Quetlet’s Index

It is an estimate of overall body fat distribution. According to the Centers for Disease Control and Prevention (CDC), BMI is calculated as weight in kilograms divided by the square of the height in meters (kg/m^2^). All the measurements were taken by using the appropriate standard technique (Table 2) [10,11].

**Table 2:** WHO Asian BMI chart

BMI classification	Percentile
Underweight	< 18.5 kg/m^2^
Normal	18.5-22.9 kg/m^2^
Overweight	23.0-24.9 kg/m^2^
Obese I	≥25.0-29.9 kg/m^2^
Obese II	≥30 kg/m^2^

Waist-Hip Ratio (WHR):

The waist circumference is measured at the approximate midpoint between the lower margin of the last palpable rib and the top of the iliac crest. The hip circumference measurement is taken around the widest portion of the buttocks. High WHR (calculated by dividing waist circumference by hip circumference) indicated abdominal fat accumulation [12]. The cut-off used for WHR for Asian adults is shown in Table 3.

**Table 3:** Cut-off for waist-hip ratio for Asian adults

Indicator	Cut off points	
	MALE	FEMALE
Normal WHR	<0.90	<0.85
High WHR	≥0.90	≥0.85

## Results

**Figure 2:**
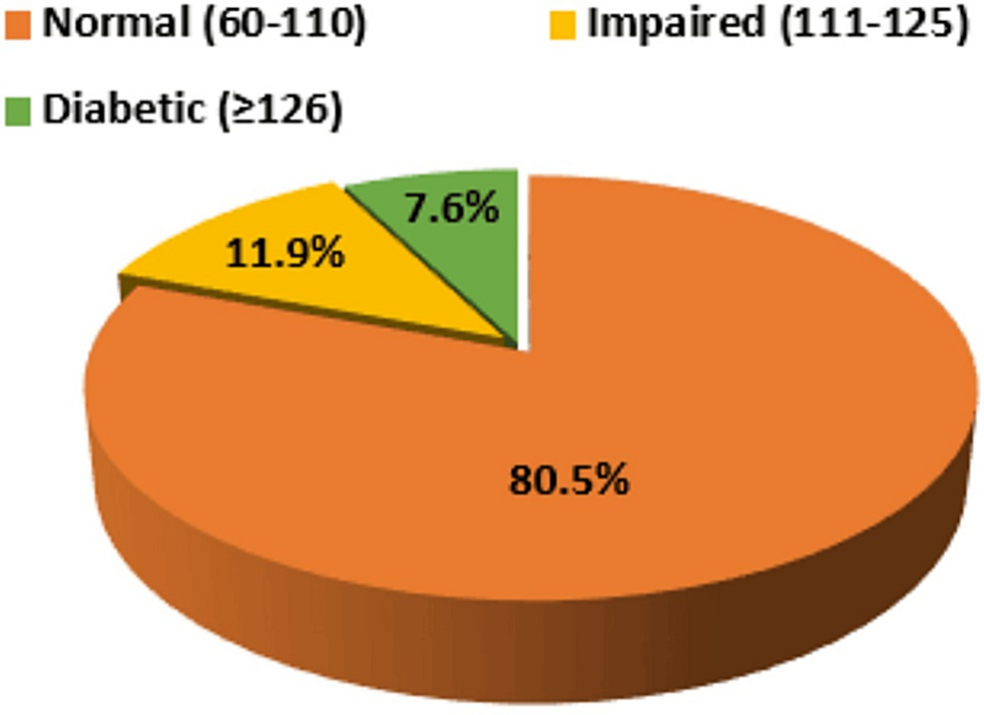
Distribution of undiagnosed diabetes mellitus among study population in relation to blood sugar level

Of the total study population, 80.5% had a fasting blood glucose level within the normal range whereas 11.9% had impaired fasting blood glucose levels. Of the study participants, 7.6% were undiagnosed diabetics with increased blood sugar levels (≥ 126 mg/dL) and were unaware of their diabetic status (see Figure 2).

In the study population, 59.4% of individuals with diabetes were aged 35-49 years, indicating an early onset of type 2 diabetes mellitus among the study participants. The majority of the subjects were females (68.7%) and married (65.6%), 71.9% engaged in either no or mild physical activity, 81.2% had a high waist-hip ratio, 21.9% were overweight, and 56.2% were classified as obese (Table 4).

**Table 4:** Baseline sociodemographic profile of study subjects of intervention group (n = 32)

Socio-demographic factors		Number	Percentage
Age Group	< 35 years	3	9.4
	35–49 years	19	59.4
	≥ 50 years	10	31.2
Gender	Male	10	31.3
	Female	22	68.7
Type of Family	Nuclear	18	56.3
	Joint	5	15.6
	Three-generation	8	25.0
	Broken/ single member	1	3.1
SLI (Standard of Living Index)	Low	0	0.0
	Medium	8	25.0
	High	24	75.0
Marital Status	Married	21	65.6
	Unmarried	3	9.4
	Widow/widower	7	21.9
	Divorced	1	3.1
Physical Activity	Vigorous	0	0.0
	Moderate	9	28.1
	Mild	14	43.8
	No activity	9	28.1
Waist–Hip Ratio	Normal	6	18.8
	High	26	81.2
BMI (Body Mass Index)	Normal	7	21.9
	Over-weight	7	21.9
	Obese Class-I	6	18.7
	Obese Class-II	12	37.5
TOTAL		32	100.0

Mean values of different anthropometric variables were measured at baseline and after three months of lifestyle modification intervention. We assessed differences in pre- and postintervention values of different anthropometric variables using the paired t-test. Overall, improvement was seen in the mean anthropometric measurements from pre- to postintervention values. There was an overall reduction in weight (66.21 ±1 2.97 to 63.18 ± 11.48), waist circumference (96.21 ± 13.01 to 91.77 ± 11.82), hip circumference (105.16 ± 11.91 to 103.58 ± 10.88), waist-hip ratio (0.91 ± 0.09 to 0.88 ± 0.08), and body mass index (BMI) (27.48 ± 6.04 to 26.18 ± 5.30). Although there was improvement in the overall means, the paired t-test showed that the differences in pre- and postintervention values of various anthropometric variables were statistically insignificant (P > 0.05) (Table 5).

**Table 5:** Pre- and Postintervention changes in anthropometric measurements Mean ± SD: Mean ± Standard Deviation of the given mentioned variables.

Variables	Preintervention	Postintervention	t-value (paired t-test)	P-value
	Baseline Mean ± SD	After 3 Months Mean ± SD		
Weight	66.21 ± 12.97	63.18 ± 11.48	0.9895	> 0.05
Waist Circumference	96.21 ± 13.01	91.77 ± 11.82	1.4288	> 0.05
Hip Circumference	105.16 ± 11.91	103.58 ± 10.88	0.5540	> 0.05
Waist–Hip Ratio	0.91 ± 0.09	0.88 ± 0.08	1.4093	> 0.05
Body Mass Index (BMI)	27.48 ± 6.04	26.18 ± 5.30	0.9151	> 0.05

We assessed differences in pre- and postintervention glycemic values using the paired t-test. There was significant improvement in the mean glycemic values of fasting blood sugar (180.19 ± 55.81 to 152.56 ± 45.74) and glycosylated hemoglobin levels (8.61 ± 1.97 to 6.68 ± 1.67) from baseline to 3 months follow-up with an intervention of lifestyle modification. The differences in pre- and postintervention mean values of fasting blood sugar and glycosylated hemoglobin levels were statistically significant (P < 0.05) (Table 6).

**Table 6:** Pre- and postintervention changes in glycemic values Mean ± SD: Mean ± Standard Deviation of the given mentioned variables.

Variables	Preintervention	Postintervention	t-value (paired t-test)	P-value
	Base Line Mean ± SD	After 3 Months Mean ± SD		
Fasting Blood Sugar	180.19 ± 55.81	152.56 ± 45.74	2.1660	< 0.05
Glycosylated Hemoglobin (HbA1c)	8.61 ± 1.97	6.68 ± 1.67	3.2417	< 0.05

## Discussion

In the present study, we observed that 80.5% of the study population had fasting blood glucose levels within the normal range whereas 11.9% had impaired fasting glucose. The findings were consistent with the studies conducted by Ravikumar et al. [13], Subramani et al. [14], Agarwal et al. [15], Anusuya et al. [16], Chaturvedi et al. [17], and Mohammad et al. [18], in which the prevalence of undiagnosed diabetes was 6.3%, 11.1%, 8.9%, 10.3%, 14.9%, and 9.9%, respectively. However, the prevalence was less than that reported by Dudeja et al. [6] and Bala et al. [19], who reported undiagnosed diabetes prevalence at 26.4% and 21.3%, respectively. The highest prevalence was reported by Namdev et al. [20] at 76%.

In the present study, all the recently diagnosed diabetics were subjected to an intervention of lifestyle modification for a period of three months, and the mean anthropometric variables and glycemic values were compared with the baseline levels. At the end of the intervention, significant improvement was seen in the mean glycemic values of blood sugar fasting (180.19 ± 55.81 to 152.56 ± 45.74) and HbA1c percentage (8.61 ± 1.97 to 6.68 ± 1.67) from baseline to three-month follow-up with an intervention of lifestyle modification. The overall means showed improvement; however, the differences in pre- and postintervention values of different anthropometric variables when assessed using the paired t-test were statistically insignificant (P > 0.05). Sukla et al. [21] reported nearly similar results, including significant differences in fasting blood sugar levels before and after the intervention. Although weight and waist circumferences were reduced among the study participants, the association was not statistically significant.

Pot et al. [22] reported significantly lower fasting glucose levels at six months (-1.2 ± 2.6 mmol/L, P = 0.001). Additionally, body weight decreased by 4.9 ± 5.1 kg (P < 0.001), waist circumference decreased by 9.4 ± 5.0 cm (P < 0.001), and BMI was lower by 1.70 ± 1.69 kg/m^2^ (P < 0.001) at six months compared with baseline. Furthermore, participants showed significantly lower Hb1Ac levels at six months (53.2 ± 12.5 mmol/mol) compared with baseline (58.3 ± 12.0 mmol/mol; P < 0.001).

Kumari et al. [3] in Delhi reported significant improvement in fasting blood sugar (175.5 ± 32.3 to 144.7 ± 17.6), postprandial blood sugar (275.5 ± 61.3 to 199.0 ± 48.3), and HbA1c percentage (9.3 ± 1.5 to 8.4 ± 1.3) in the interventional group when they received lifestyle modification counseling using the lifestyle intervention holistic model. Therefore, counseling for lifestyle modification is an effective and noninvasive method for managing glycemic control in type 2 diabetes mellitus.

Limitations of the study included the strict compliance with the routine chart given as participants needed daily one-to-one contact sessions which were not possible.

## Conclusions

The study conducted in the urban field practice area of the Department of Community Medicine in Meerut, involving 503 adults aged 30 years and above, highlights the significant impact of lifestyle modification on glycemic control in diabetic patients. With the implementation of lifestyle changes, mainly increased physical activity and adherence to a healthy diet, we observed a substantial improvement in mean glycemic values, including fasting blood sugar and HbA1C percentage, over a period of three months.

These findings emphasize the importance of incorporating physical activity as a crucial component in managing blood sugar levels among individuals with diabetes. The inclusion of rhythmic daily physical activities such as brisk walking, yoga, and pranayama can not only enhance glycemic control but also help prevent diabetes-related complications and will eventually make the slogan “Move For Health” a reality.
